# Transcriptomic and biochemical insights into LOX pathway aroma biosynthesis during ripening of *Ziziphus jujuba* Mill. *cv.* Li

**DOI:** 10.1016/j.fochms.2025.100347

**Published:** 2025-12-30

**Authors:** Lina Wang, Qiaoling Liu, Chuan Chen, Rongfa Guan, Peilong Sun

**Affiliations:** a College of Food Science and Technology, Zhejiang University of Technology, Hangzhou 310014, China; b School of Digital Economics, Wenzhou Vocational College of Science and Technology, Wenzhou 325000, China; c Moganshan Institute ZJUT, Kangqian District, Deqing 313200, China

**Keywords:** Volatile organic compounds, Odor activity value, Fruit ripening, Metabolic engineering

## Abstract

Aroma is a critical determinant of fruit quality, largely synthesized through the lipoxygenase (LOX) pathway. We hypothesized that the dynamic regulation of the LOX pathway during jujube fruit ripening governs the distinct evolution of its key aroma compounds. This study used HS-SPME coupled with GC–MS to profile aroma compounds in jujube during ripening. Semi-quantitative analysis revealed hexanal (1160–1870 μg/kg) and (E)-2-hexenal (1470–3180 μg/kg) as the most abundant aldehydes, followed by benzaldehyde and (E)-2-pentenal. Odor activity value (OAV) analysis identified hexanal, (*E*)-2-hexenal, and (*E*)-2-nonenal as the key aroma compounds. LOX enzyme activity increased from 277 U/g to 711 U/g during ripening, while ADH and AAT activities showed fluctuating trends. Transcriptome analysis revealed 12 candidate transcripts involved in aroma synthesis, with multivariate statistical analysis demonstrating coordinated changes in gene expression associated with volatile accumulation. Our findings verify the hypothesis that LOX pathway regulation drives aroma evolution during jujube ripening and provide a genetic foundation for targeted quality improvement in jujube fruits.

## Highlights

• Identified hexanal, (E)-2-hexenal, and (E)-2-nonenal as the key aroma compounds.
• LOX activity increased 2.6-fold during ripening while ADH activity remained low.
• Revealed a metabolic bottleneck at the ADH step, explaining the lack of alcohols.
• Transcriptome analysis revealed 12 candidate transcripts regulating volatiles.
• Multivariate analysis linked gene expression patterns with volatile accumulation.

## 1 Introduction

Jujube (*Ziziphus jujuba* Mill.), a member of the Rhamnaceae family indigenous to China, has been cultivated for over 4000 years. It is an economically important crop and has been introduced into more than 40 countries worldwide (Liu et al., 2014). Jujube is rich in nutrients (vitamins, minerals, amino acids, fatty acids) and bioactive phytochemicals (phenolics, polysaccharides) (Chen et al., 2018; Song et al., 2019; Zhu et al., 2024). Furthermore, in the food industry, jujube is utilized as food additive and flavoring agent, an application attributed to its broader potential as a functional ingredient (Rashwan et al., 2020) and its distinctive aroma profile(Chen et al., 2018). Therefore, elucidating the chemical and genetic basis of jujube aroma formation is essential for targeted quality improvement.

Aroma is an important feature that influences the fruit quality. In many fruits, the lipoxygenase (LOX) pathway is a primary source of volatile compounds. This pathway converts polyunsaturated fatty acids like linoleic and linolenic acid into volatile C6 and C9 aldehydes (e.g., hexanal and (*E*)-2-hexenal). These aldehydes form the foundational precursor pool for biosynthesis of fatty acid-derived aroma compounds. They can be further converted to corresponding alcohols and esters by downstream enzymes such as hydroperoxide lyase (HPL), alcohol dehydrogenase (ADH), and alcohol acyltransferase (AAT) (Vincenti et al., 2019; Yun et al., 2022).In jujube, aldehydes such as hexanal, (*E*)-2-hexenal, and (*E*)-2-nonenal are consistently reported as dominant volatiles (Chen et al., 2018; Hernández et al., 2016; Wang et al., 2019).Notably, however, LOX-derived alcohols and esters which are commonly abundant in other fruits(Chen et al., 2020; Gu et al., 2022; Zou et al., 2018), are scarcely reported in jujube(Song et al., 2019; Wang et al., 2019). This compositional disparity suggests a potential bottleneck in the LOX pathway, possibly at the aldehyde-to-alcohol conversion step mediated by ADH.

Transcriptome analysis provide a chance to reveal the mechanism of compounds synthesis in plants (Shen et al., 2022). In fruits such as watermelon, blueberry, and grape, transcriptomic approaches have identified key genes including ADH, LOX, and regulatory transcription factors, that control the synthesis of aroma compounds and their dynamic changes during ripening or postharvest storage (Gong et al., 2021; Li et al., 2023; Yuan et al., 2020). Similarly, in tomato, reduced *ADH2* expression leads to lower alcohol levels, highlighting the transcriptional regulation of the LOX pathway (Zou et al., 2018). Although LOX and HPL genes including *ZjLOX3*, *ZjLOX4* and *ZjHPL1* are functionally linked to (*E*)-2-hexenal synthesis in *Ziziphus jujuba* Mill. cv. Dongzao and Junzao (Yue et al., 2022), a systematic transcriptomic investigation into the coordinated regulation of the entire LOX pathway across ripening stages remains lacking.

To address this knowledge gap, we hypothesize that the predominance of aldehydes and the general absence of LOX-derived alcohols and esters in jujube cv. Li are due to constrained ADH activity and/or gene expression during ripening, leading to an accumulation of upstream aldehydes and a blockage in downstream ester formation. To test this hypothesis, the aroma compounds were identified and semi-quantified volatile compounds across ripening stages using HS-SPME-GC–MS, focusing on odor-active compounds via OAV analysis. Then, the activity of key enzymes (LOX, ADH and ATT) which involved in LOX pathway were evaluated. Furthermore, transcriptome sequencing to identify candidate transcripts which regulated aroma synthesis was conducted. Finally, multivariate integration analysis was performed to correlate gene expression with volatile and enzyme activity dynamics. This study aims to delineate the biochemical and genetic framework of aroma development in jujube, providing a foundation for targeted quality improvement.

## 2 Material and methods

### 2.1 Sample collection

Fruits of *Ziziphus jujuba* Mill. *cv.* Li were harvested from National Jujube Germplasm Resources Center in Shanxi Province, China. Fruits at three ripening stages including white stage (LS1), half-red stage (LS2), and full-red stage (LS3) were harvested based on visual color. For each stage, three independent biological replicates were collected, with each replicate consisting of a pool of 20 fruits. All selected fruits were in the similar size and free from physical damage. The fruits were transported to the laboratory within 4 h of harvest and stored at −80 °C for subsequent analysis to minimize enzymatic degradation of volatile compounds. Additionally, a separate batch of LS3 fruits was stored at ambient temperature (25 °C) for 15 days to simulate a post-ripening stage (LS4) before being processed and stored at −80 °C.

### 2.2 Chemicals

N-alkane standard (C8-C20) was obtained from Sigma-Aldrich, (St. Louis, MO., USA); n-hexane, 6-methyl-5-heptene-2-ol standard (> 99 %) were purchased from Xinyang Zhongjian Metrology Biological Co., Ltd. (Xinyang, Henan, China); lipoxygenase (LOX) activity detection kit, alcohol dehydrogenase (ADH) activity detection kit and alcohol acyltransferase (AAT) kit in plants were bought from Beijing Suolaibao Technology Co., Ltd. (Beijing, China).

### 2.3 Volatile compounds identification and quantification

The analysis of volatile compounds was performed based on the method of Wang et al. (2019) with modifications. Briefly, the 20 jujube fruits (seeds off) were homogenized in liquid nitrogen for each biological replicate without seeds. Then, 3.00 g fruit sample from each biological replicate of different ripening stages were weighed for the analysis. The tissue was then transferred to a 20 mL headspace vial which was immediately sealed. The sample was equilibrated at 50 °C in a water bath for 15 min. A DVB/CAR/PDMS fiber (50/30 μm, Supelco, Bellefonte, PA, USA) was used for headspace solid-phase microextraction (HS-SPME). After equilibration, the fiber was inserted into the sample vial and exposed to the headspace to adsorb volatile compounds. The extraction was conducted at 50 °C in a water bath for 30 min, with the fiber tip maintained approximately 10 mm above the sample tissue. Following extraction, the fiber was removed from the vial and immediately inserted into the GC injection port for desorption and analysis. For semi-quantification, 10 μL of an internal standard solution (6-methyl-5-hepten-2-ol in n-hexane, at a final concentration of 50 mg/kg in the sample) was added to the sample prior to SPME extraction. The n-hexane solvent was allowed to evaporate completely before vial sealing. Data are expressed as semi-quantitative concentrations relative to the internal standard (μg ISE/kg).

DSQ II single-quadrupole gas chromatography–mass spectrometry (Thermo Scientific, USA) coupled with DB-5 column (60 × 25 mm, 0.25 μm, Agilent Technologies, Santa Clara, CA, USA) was used for volatile compounds analysis. Conditions for the GC were as follows: desorption 3 min, inlet temperature of 250 °C, transfer line temperature of 250 °C, initial temperature of 40 °C, hold for three minutes, then increased the temperature by 10 °C/min to 200 °C, hold for five minutes, and finally increased the temperature by 10 °C /min to 240 °C, hold for ten minutes. Electron ionization (EI) mode on the mass spectrometry detector, an ion source temperature of 250 °C, an electron energy setting of 70 eV, and a mass spectrometry scan range of 35–500 *m/z*. Compound identification was performed by comparing mass spectra with the NIST (v2.0) library and by calculating retention indices (RIs) using a homologous series of n-alkanes (C8-C20). Each biological replicate was analyzed once. The mean and standard deviation reported are derived from the three biological replicates (*n* = 3).

The RI calculation formula is given below:

(1) $$\mathrm{RI}=100\times [n+\left(N-n\right)\times \frac{\log t-\log t_{n}}{\log t_{N-\log t_{n}}}$$

where t is the retention time for the detected compound, t_n_ is the retention time for the alkane standard eluted prior to the compound, n is the number of carbons of that alkane standard, and t_N_ is the retention time for the alkane standard substance eluted following the compound.

### 2.4 Odor activity value calculation

(2) $$\mathrm{OAV}=\frac{\mathrm{Ci}}{\mathrm{OTi}}$$

The amount of C_i_ -aroma compounds (μg/kg) and OT_i_-the threshold for this compound in water (μg/kg) are given in the formula.

### 2.5 LOX pathway key enzyme activity determination

UV spectrophotometry was used to measure the activity of the key enzymes LOX, ADH, and AAT. The precise procedures were exactly followed the instructions of the kits.

### 2.6 RNA extraction, quantification and sequencing

TRIzol reagent (Invitrogen, Carlsbad, CA, USA) was used to extract total RNA, and the procedure was followed the kit protocol. NanoDrop 2000 spectrophotometer (Thermo Scientific, Wilmington, DE, USA) was used to evaluate purity and quantification of RNA. RNA integrity was assessed using the Agilent 2100 Bioanalyzer (Agilent Technologies, Santa Clara, CA, USA). Then the libraries were constructed using VAHTS Universal V6 RNA-seq Library Prep Kit followed the manufacturer's instructions. The libraries were sequenced on a llumina Novaseq 6000 platform and 150 bp paired-end reads were generated. The transcriptome sequencing and analysis were conducted by OE Biotech Co., Ltd. (Shanghai, China).

### 2.7 Transcriptome data analysis

Raw reads in FASTQ format were filtered to remove the low-quality reads and obtained clean reads. Then the clean reads were mapped using HISAT2 to the reference genome. Fragments per kilobase of transcript sequence per millions base pairs sequenced (FPKM) of each gene was calculated and the reads counts were obtained by HTSeq-count. Differential expression genes (DEGs) were performed by DESeq2 with *q*-value <0.05 and fold change>2.0 or fold change<0.5. Gene ontology (GO) and Kyoto encyclopedia of genes and genomes (KEGG) enrichment analysis of DEGs were processed by R (v 3.2.0)(Zhang et al., 2020). Candidate transcripts were selected based on differential expression analysis, and functional annotation related to known aroma synthesis pathways.

### 2.8 Statistics

All measurements (volatile compounds, enzyme activities) were performed once per biological replicate. Statistical analyses were based on three independent biological replicates (*n* = 3). Data are presented as mean ± standard deviation of these biological replicates. Significance of differences among ripening stages was determined by one-way analysis of variance (ANOVA) followed by Duncan's multiple range test using SPSS 26.0 software (IBM Corp., Chicago, IL, USA). Differences were considered significant at *p* < 0.05. PCA analysis was generated by JMP Pro 14, the other multiple statistics and the co-expression correlation analysis were run by the website tools of OE Biotech Co., Ltd. (Shanghai, China).

## 3 Results and discussion

### 3.1 Volatile compounds identification and semi-quantification

Volatile compounds as secondary metabolites provide unique flavor of fruits (Liu et al., 2021). In this study, total 24 compounds were identified across four ripening stages (Table 1). It is important to note that, in the absence of authentic standards for all compounds, the concentrations reported are semi-quantitative. They are based on the response factor of the internal standard 6-methyl-5-hepten-2-ol, and therefore represent relative abundances. Aldehydes dominated the volatile profile of jujube cv. Li, with the cumulative content of detected compounds generally increasing during ripening and peaking at LS4. Notable exceptions to this trend were (*E*)-2-pentenal, octanoic acid octyl ester, ethyl isocholate, and hexanoic acid.

**Table 1:** The semi-quantification contents of volatile compounds in *Ziziphus jujuba* Mill. *cv.* Li from different ripening stages, ISE μg/kg.

No.	Aroma components	Reference RI value	RI	*Ziziphus jujuba* Mill. *cv.* Li μg/kg			
				LS1	LS2	LS3	LS4
	Aldehydes						
1	Acetaldehyde	<500	<500	97.0 ± 4.0 a	nd	33.0 ± 14.0 b	120 ± 30 a
2	*(E)*-2-pentenal	699	683	150 ± 3 c	110 ± 58 c	430 ± 23 a	280 ± 24 b
3	Pentanal	690	691	24.0 ± 11.0 a	64.0 ± 5.0 a	53.0 ± 17.0 a	70.0 ± 5.7 a
4	Hexanal	799	800	1160 ± 140 c	1360 ± 140 b	1650 ± 110 b	1870 ± 200 a
5	(*E*)-2-hexenal	858	859	2310 ± 190 b	1470 ± 70 c	2240 ± 19 b	3180 ± 580 a
6	Heptanal	903	900	nd	77.0 ± 21.0	nd	nd
7	*(E, E)*-2,4-Hexadienal	910	907	44.0 ± 13.0b	52.0 ± 11.0 b	34.0 ± 13.0 b	370 ± 19 a
8	Benzaldehyde	966	968	300 ± 15 b	660 ± 61 b	550 ± 136 b	4590 ± 380 a
9	*(E)*-octenal	1060	1059	84.0 ± 8.5 b	110 ± 3 ab	140 ± 28 a	120 ± 20 a
10	*(E)*-2-nonenal	1162	1163	46.0 ± 1.1 b	54.0 ± 16 b	nd	600 ± 34 a
11	Decanal	1202	1206	nd	nd	nd	140 ± 28

	Ester						
1	Nonanoic acid pentylester	–	1704	56.0 ± 25 c	230 ± 42 b	nd	360 ± 68 a
2	Octanoic acid octyl ester	1779	1780	290 ± 31a	52.0 ± 5.0 b	43.0 ± 13.0 b	62.0 ± 10.8 b
3	Ethyl isocholate	–	1934	26.0 ± 2.9 b	220 ± 46 a	27.0 ± 1.0 b	nd

	Ketone						
1	1-pentene-3-one	680	640	38.0 ± 4.0 c	71.0 ± 4.0 b	36.0 ± 2.0 c	84.0 ± 10.0 a
2	Geranyl acetone	1455	1456	nd	nd	55.0 ± 12.0 b	110 ± 11 a

	Acids						
1	Hexanoic acid	1015	975	nd	nd	310 ± 3 a	190 ± 22 b
2	Nonanoic acid	1271	1259	nd	nd	nd	nd
3	Decanoic acid	1371	1357	nd	43.0 ± 2.0 b	51.0 ± 18.0 b	1210 ± 25 a
4	Lauric acid	1567	1552	nd	nd	100 ± 6 a	120 ± 33 a
5	*(Z)*-7-tetradecenoic acid	–	1747	nd	nd	nd	270 ± 41
6	Tetradecanoic acid	1751	1755	120 ± 12 b	24.0 ± 3 c	73.0 ± 24.0 bc	280 ± 49 a

	Alcohols						
1	1-penten-3-ol	686	639	36.0 ± 11.0	nd	nd	nd
2	Benzyl alcohol	1032	1037	nd	nd	30.0 ± 6.0 b	220 ± 24 a

	Other						
1	beta-Cadinene	1529	1548	nd	220 ± 11b	nd	4280 ± 80 a

Note: “nd” means not detected, data was expressed as mean value ± standard deviation, different lowercase letter after the data in the same row means significant difference (*p* < 0.05).RI was calculated based the experiment test. Reference RI was cited from NIST (National Institute of Standards and Technology Standard Reference Database, Gaithersburg, USA).

Aldehydes, primarily derived from unsaturated fatty acid oxidation, constituted the principal aroma contributors in jujube. (*E*)-2-pentenal, pentanal, hexanal, (*E*)-2-hexenal, (*E, E*)-2,4-hexadienal, benzaldehyde, (*E*)-octenal were persistently detected throughout all ripening stages. Hexanal (1160–1870 μg/kg) and (*E*)-2-hexenal (1470–3180 μg/kg) were the most abundant aldehydes, with the concentrations generally increasing with the maturity stage. This pattern aligns with previous observations in guava fruits, where both compounds increased from immature to mature stages (Moon et al., 2018). Previous studies have consistently identified aldehydes as the predominant volatile compounds in jujube fruits (Wang et al., 2019). Chen et al. reported that hexanal, (*E*)-2-hexenal, (*Z*)-2-heptenal, benzaldehyde and (*E*)-2-nonenal were the main compounds in ten varieties of jujube (Chen et al., 2018). Similarly, Wang et al. (2019) identified hexanal, (*E*)-2-hexenal and benzaldehyde were major volatile compounds in jujube. In this study, (*E*)-2-nonenal was the main aldehyde at LS4, while (*Z*)-2-heptenal was not detected, possibly reflecting differences in maturity stages and cultivars. The content of (*E*)-2-hexenal was the most abundant aldehydes, it was demonstrated by previous reports (Chen et al., 2018; Song et al., 2019; Wang et al., 2019). The content of (*E*)-2-pentenal was increased from LS1 to LS3, however, it was decreased at LS4. Pentanal content remained relatively stable across all stages without significant differences. Heptanal was only detected at LS2 and (*E*)-2-nonenal, though generally increasing with maturity, was not detected at LS3. Decanal was only found at LS4, this may be affected by post-harvest ripening. Furthermore, the concentration of hexanal, (*E*)-2-hexenal, (*E,E*)-2,4-hexadienal, benzaldehyde all increased along with the maturity stages, it was similar as Song's results (Song et al., 2019). This pattern contrasts with passion fruit, where aldehyde levels decrease during ripening (Liu et al., 2014).

Esters are generally derived from fatty acids and amino acids pathways (Li et al., 2014). Three esters were detected in cultivar Li. In other fruits, the esters content were increased during ripening, as observed in grapes (Cabernet Gernischet, Cabernet Franc and Merlot) (Gu et al., 2022) and cape gooseberry (Espino-Díaz et al., 2016), where ethylene driven respiration enhances ester production. Ketones displayed limited diversity, with two ketones (1-penten-3-one and geranyl acetone) were detected in Li jujube, and geranyl acetone was only found in LS3 and LS4, while 1-pentene-3-one was found at all the stages. In cape gooseberry, 1-pentene-3-one was showed higher content in greenish-yellow and yellow stages. Some short chain acids were produced by β-oxidation of the long chain fatty acids, including acetic acid, and hexanoic acid (Espino-Díaz et al., 2016). Most of acids were not found in LS1, this was the same as acids at the first stage in the Rose roxburghii. Hexanoic acid and lauric acid were not found even at LS2. Decanoic acid was detected from LS2 to LS4, and its amount was increased, reaching the highest content at LS4 (1210 μg/kg). 1-penten-3-ol was only found at LS1 stage, while benzyl alcohol was detected at LS3 and LS4 and increased with the ripening. Gu et al. (2022) found that benzyl alcohol was increased with the ripening in all the grape cultivars in their detection. In Rose roxburghii, 1-hexanol and (*E*)-3-hexen-1-ol were detected, indicating the conversion of hexanal to1-hexanol by ADH (Xu et al., 2024). Unfortunately, these two alcohols were not detected in this study, it may relate to the low ADH activity.

Our semi-quantitative profiling revealed a clear ripening-dependent accumulation of C6 and C9 aldehydes, particularly hexanal and (*E*)-2-hexenal, in jujube cv. Li. This pattern is consistent with the activation of the LOX pathway observed in other fruits like guava and pear (Moon et al., 2018; L. Zhang et al., 2018). The distinct dominance of aldehydes over alcohols and esters, however, sets jujube apart from many climacteric fruits. The surge in (E)-2-nonenal at the post-ripening stage (LS4) is particularly noteworthy. As a C9 aldehyde derived from the 9-LOX pathway, its accumulation suggested a shift in LOX regiospecificity or substrate availability during late maturation and storage, a phenomenon less commonly reported in jujube. The detection of benzaldehyde exclusively in the later stages aligns with its known biosynthesis from phenylalanine via the phenylpropanoid pathway, which is often developmentally regulated.

### 3.2 OAV analysis of volatile compounds

Volatile compounds with OAV greater than 1 were considered as an aroma compound due to the contribution to the aroma of samples (Guth, 1997), where higher OAV indicate greater aroma contributions (Zhang et al., 2019). Based on Table 2, 18 compounds exhibited OVA >1 at the LS1 developmental stage, with hexanal (OAV: 259), (*E*)-2-hexenal (OAV: 136), (*E*)-2-octenal (OAV: 21), 1-Penten-3-one (OAV: 29) and (*E*)-2-nonenal (OAV: 229) demonstrating dominant contributions. At LS2 stage, OAV of 10 compounds were larger than 1, with hexanal (OAV: 302), (*E*)-2-hexenal (OAV: 87), heptanal (OAV: 26), (*E*)-2-octenal (OAV: 28), (*E*)-2-nonenal (OAV: 272), 1-penten-3-one (OAV: 55) as primary aroma compounds. The first two stage (LS1, LS2) shared similar aromatic characteristics dominated by green and fruity notes. At LS3, hexanal (OAV: 367), (*E*)-2-hexenal (OAV: 132), (*E*)-2-octenal (OAV: 19), 1-penten-3-one (OAV: 28) and lauric acid (OAV: 52) became prominent contributors. While at LS4 stage, hexanal (OAV: 416), (*E*)-2-hexenal (OAV: 187), (*E*)-2-nonenal (OAV: 3021), 1-penten-3-one (OAV: 65), lauric acid (OAV: 60) were the main aroma compounds. Fatty aroma was appeared as one of the main aroma compounds, this may involve in the ripening process and the metabolites pathway. While consumers may generally prefer the characteristic green and fruity notes associated with hexanal, (*E*)-2-hexenal, (*E*)-2-octenal and 1-penten-3-one (Liu et al., 2022; Wojdyło et al., 2016). Our findings demonstrated a ripening dependent dominance of C6-C10 aldehydes. This progressive increase in aldehyde contributions, particularly the remarkable OAV of 3021 for (*E*)-2-nonenal at LS4, confirms their critical role in jujube fruit aroma development (Wang et al., 2016; Zhu & Xiao, 2018) (See Table 2).

**Table 2:** The odor activity value of aroma compounds of *Ziziphus jujuba* Mill. *cv.* Li.

NO.	Aroma compounds	Threshold value (μg/kg)	Aroma characteristics	LZ			
				LS1	LS2	LS3	LS4
1	Acetaldehyde	15.0	fermentation	7	–	2	8
2	(*E*)- 2-Pentenal	1500	wood	–	<1	<1	<1
3	Pentanal	12.0	fatty	2	5	4	6
4	Hexanal	4.50	grass	259	302	367	416
5	(*E*)-2-Hexenal	17.0	grass	136	87	132	187
6	Heptanal	3.00	fatty	–	26	–	–
7	(*E, E*)-2,4-Hexadienal	60.0	fatty	<1	<1	<1	<1
8	Benzaldehyde	350	wood and violet	<1	1	1	1
9	(*E*)-2-Octenal	4.00	floral, fruity, fatty	21	28	19	31
10	(*E*)-2-Nonenal	0.230	cucumber	229	272	–	3021
11	Ethyl isocholate	15.0	tobacco	2	17	2	–
12	Nonanoic acid, pentyl ester	57.0	nutty, fatty and almond	<1	5	–	11
13	1-Penten-3-one	1.30	fish, ether	29	55	28	65
14	Geranyl acetone	60.0	incense of magnolia	–	–	<1	2
15	Hexanoic acid	3000	fatty odor, slightly sour, sweet	–	–	<1	<1
16	Lauric acid	2.00	fatty	–	–	52	60
17	1-Penten-3-ol	400	fruit	<1	–	–	–

Note: “-” means the compounds were not detected at the ripening stage; threshold values are from the Compilation of Olfactory Thresholds for Compounds, Second Edition.

### 3.3 LOX pathway key enzyme activity determination

**Fig. 1:**
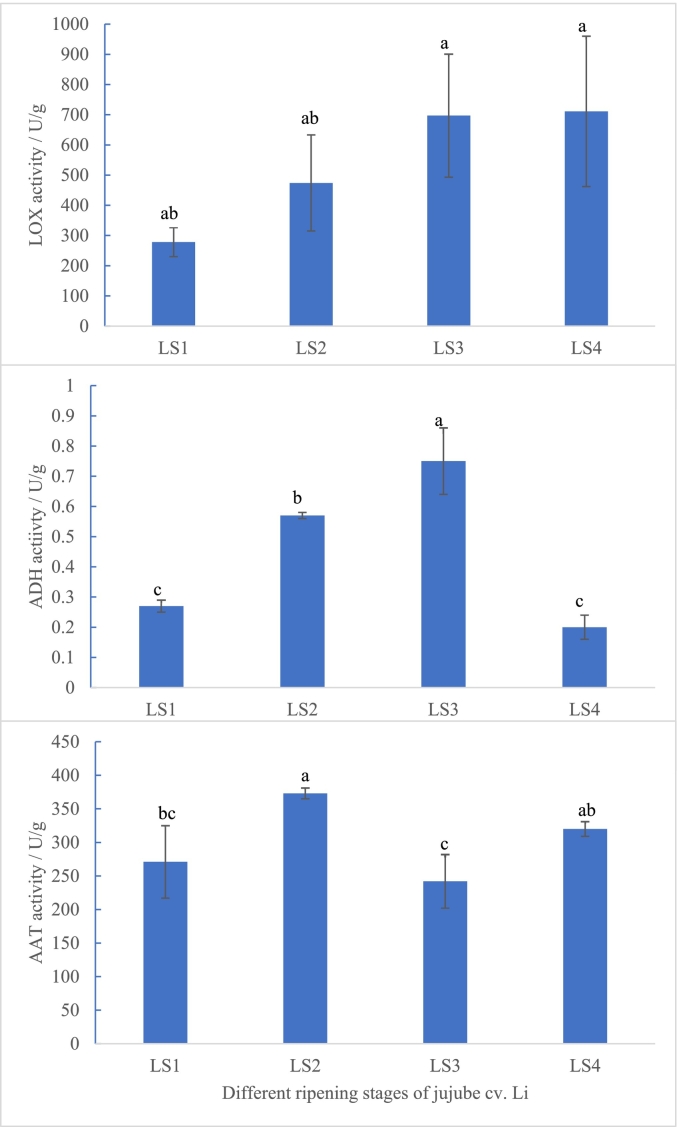
LOX activity (A), ADH activity (B) and AAT activity (C) at different ripening stages of *Ziziphus jujuba* Mill. *cv.* Li (different lowercase of letters on the top of column in the figure means significant difference, *p* < 0.05).

The aroma compounds in jujube fruits are synthesized through LOX pathway, C18:2 and C18:3 fatty acids were oxygenated by LOX, followed by cleavage by hydroperoxide lyase (HPL) to produce C6 or C9 aldehydes, which can be further converted to alcohols and esters by ADH and AAT, respectively (Vincenti et al., 2019). All illustrated in Fig. 1 A, LOX activity in jujube Li was increased with ripening (277 U/g to 711 U/g), paralleling the accumulation pattern of hexanal. Notably, (*E*)-2-hexenal, another main aroma compound, showed progressive enrichment along with the ripening stages except at LS2. These observation confirmed the predominance of the 13-hydroperoxide of LOX pathway in generating C6 aldehydes (Wu et al., 2020). However, the higher content of (E)-2-hexenal compared to hexanal suggests greater involvement of C18:3 in the metabolic pathway. It may be related to the sufficiency of C18:3 or the preference of LOX, this phenomenon consistent with findings in grape and strawberry fruits (Kalua & Boss, 2010).

Although ADH typically catalyzed aldehyde to alcohol conversion (e.g., hexanal to hexanol), no C6 alcohols were detected despite measurable ADH activity (0.35–1.78 U/g from LS1 to LS2 in Fig. 1 B). This level of ADH activity appears insufficient for alcohol accumulation, aligning with similar findings in other jujube cultivars (Chen et al., 2018; Song et al., 2019). In contrast, grapes, apples, blueberries (Yuan et al., 2020), Rose *roxburghii* (Xu et al., 2024) and tomatoes utilized this pathway effectively (Zou et al., 2018). In ‘Modesto’ and ‘Perfection’ apricots, the aldehydes was decreased during ripening with the increase of *lox* expression, because they were converted to the alcohol (González-Agüero et al., 2009), but in this study, no related alcohols were produced, the persistent aldehyde accumulation in jujube implied a unique metabolic block at the ADH step.

AAT catalyzes the substrate alcohols to produce the ester aromas in LOX pathway (Zhang et al., 2018). AAT activity increased from LS1 to LS2 (242 U/g to 373 U/g) before declining at LS3 (Fig. 1 C). Although AAT activity exceeded ADH activity throughout ripening, no corresponding esters were detected. Previous research had indicated that alcohol substrate can limited the ester production (Cano-Salazar et al., 2013). In apricot, the 1-hexanol was used as the substrates for ester formation during ripening, and it was mentioned that maybe some other critical steps involved in ester biosynthesis except the enzyme activity (González-Agüero et al., 2009). Zhang et al. (2018) observed that AAT activity in Nanguo pears treated with exogenous ATP increased at 6 days during shelf life before decreasing, this trend was similar as jujube across different ripening stages. In this study, it supported substrate-dependent regulation rather than enzymatic capacity as the limiting factor for C6 ester generation, particularly given the absence of detectable C6 alcohols in jujube fruits.

### 3.4 Transcriptome sequencing data analysis

Total RNA was extracted from each of the three biological replicates for every ripening stage (LS1-LS4). Libraries were constructed individually and sequenced, yielding one set of sequencing data per biological replicate using the Illumina platform. After filtering, the clean reads varied from 6.08 to 7.10 G, which occupied more than 95 % of valid bases. In the samples, the Q30 value was from 92.2 to 94.4 %, and the GC content was ranged from 44.2 to 44.7 %. Genome alignment of each sample was determined by comparing the reads to the reference genome; the alignment rate ranged from 87.2 to 91.8 %. (See Table 3) To understand the differences between different maturity stage, gene expression profiles were further analyzed through FRKM value. DEGs were investigated specific to each stage.

**Table 3:** Summary of transcriptome sequencing of jujube cv. Li at different ripening stages.

Sample	Raw Bases(G)	Clean Bases(G)	Valid Bases(%)	Q30 (%)	GC (%)
LS1–1	6.43	6.20	96.3	92.2	44.4
LS1–2	6.86	6.61	96.4	92.6	44.2
LS1–3	7.07	6.83	96.6	92.6	44.4
LS2–1	7.04	6.79	96.5	92.8	44.7
LS2–2	6.38	6.14	96.3	92.3	44.7
LS2–3	6.32	6.08	96.3	92.4	44.7
LS3–1	6.88	6.59	95.8	94.4	44.4
LS3–2	7.42	7.10	95.8	94.0	44.4
LS3–3	7.16	6.86	95.8	94.2	44.2
LS4–1	6.76	6.47	95.8	94.1	44.2
LS4–2	6.53	6.25	95.7	94.1	44.6
LS4–3	6.83	6.54	95.7	94.2	44.4

Note: sample names LS1, LS2, LS3 and LS4 means jujube Li from S1, S2 and S3 and S4 stage respectively, and number 1, 2, 3 after dash line means the numbers of repeat tests.

### 3.5 Analysis of genes involved in aroma synthesis

To identify key genetic determinants of aroma formation, we focused on candidate transcripts implicated in the biosynthesis of volatile compounds through fatty acids metabolism, amino acids metabolism, and carbohydrate metabolism (Espino-Díaz et al., 2016). Table 4 listed the candidate transcripts potentially involved in aroma synthesis metabolisms, selected based on differential expression analysis and functional annotation related to known aroma biosynthesis pathways.

**Table 4:** Candidate transcripts for aroma synthesis in fresh jujube fruits at different maturity stages.

Enzyme	EC	Gene ID	Regulation					
			LS2vsLS1	LS3vsLS1	LS4vsLS1	LS3vsLS2	LS4vsLS2	LS4vsLS3
Acetyl-CoA acyltransferase	EC:2.3.1.9	LOC107415786	Up	Up	Up	Up	Up	Up
Aldehyde dehydrogenase	EC:1.2.1.3	LOC107421092	Up	Up	Up	–	–	–
		LOC107428629	Up	–	–	–	Up	Up
Pyruvate kinase	EC:2.7.1.40	LOC107430461	Up	Up	Up	–	Up	Up
Lipoxygenase	EC:1.13.11.12	LOC107428338	Down	Down		Down	Down	Down
Alcohol dehydrogenase	EC:1.1.1.1	LOC107421625	Down	–	Down	Down	Down	–
		LOC107421626	Down	–	Down	Down	Down	–
		LOC107426564	Down	Down	–	–	Down	Down
phenylalanine ammonia-lyase	EC:4.3.1.24	LOC107413687	Down	Up	Up	–	–	Up
linoleate 9 s-lipoxigenase	EC:1.13.11.58	LOC107412009	Up	–	Up	–	–	Up
		LOC107411945	–	Down	–	Down	Down	Down
		LOC107423042	Down	Down	–	Down	Down	Down

note: “-” means the gene was not the differently expression gene.

As known in Table 1, the dominant aroma compounds identified were (*E*)-2-hexenal, hexanal, and benzaldehyde. (*E*)-2-hexenal and hexanal were synthesis via α-linolenic acid and linoleic acid metabolism respectively through LOX pathway (Espino-Díaz et al., 2016; Li, Qi, et al., 2022; Li, Zhao, et al., 2022). While LOX enzyme activity increased during ripening, paralleling the accumulation of hexanal, transcriptome analysis revealed downregulation of a LOX-encoding transcript (LOC107428338). This discrepancy suggested post-transcriptional regulation or compensatory mechanisms. Interestingly, (*E*)-2-hexenal content decreased at LS3 and LS2 compared to LS1, possibly reflecting LOX suppression, yet rebounded at LS4 despite persistent gene downregulation, implying the involvement of alternative regulatory pathways. Transcription factors are critical modulators of aroma biosynthesis. Previous studies have demonstrated their roles in activating *PpLOX4* (Wang, Song, et al., 2022; Wang, Zhang, et al., 2022) and *PpAAT1* (Cao et al., 2021) expression, as well as regulating *DkADH1* expression (Jamil et al., 2019)*.*The basic leucine zipper (*b*ZIP) transcription factor family had been shown to regulate fatty-acid derived volatiles in Nanguo pear (Chen et al., 2024). Therefore, there might be some transcription factors involved in the synthesis of hexanal and (*E*)-2-hexenal in jujube as well. For (*E*)-2-nonenal production, linoleate 9 s-lipoxigenase (EC:1.13.11.58) catalyzed oxygenation at the C-9 position of linoleic acid (Vincenti et al., 2019). The upregulated transcript LOC107412009 encoding linoleate 9 s-lipoxigenase (EC:1.13.11.58) at LS2, and LS4; coupled with downregulation of transcripts LOC107411945 and LOC107423042 at LS3, aligns with (*E*)-2-nonenal accumulation patterns (Table 4).

Aldehyde dehydrogenase (ALDH, EC:1.2.1.3) associated with aldehydes biosynthesis, showed upregulation through transcripts LOC107421092 (LS3 vs. LS1 and LS4 vs. LS1) and LOC107428629 (LS2 vs. LS1, LS4 vs. LS2 and LS4 vs. LS3). The NAD-depended enzyme ADH reduced the aldehyde to alcohol with a wide range of branched, linear and cyclic alcohols (Espino-Díaz et al., 2016; Vincenti et al., 2019). In jujube cv. Li, 1-penten-3-ol was only detected in LS1 stage, which might be related to the downregulation of ADH encoding transcripts (LOC107426564, LOC107421525 and LOC107421526). ADH plays an important role in volatile synthesis in other fruits such as grapes, apples, pears. The observed downregulation of *PuADHs* in the fruits (10 DAH vs 0 DAH) (Li, Qi, et al., 2022; Li, Zhao, et al., 2022), parallels our findings in jujube. Benzaldehyde was synthesis from phenylalanine by the phenylalanine ammonia-lyase (PAL), hydratase and lyase (Widhalm & Dudareva, 2015). The content of benzaldehyde increased from LS1 to LS4, with no significant differences between LS2 and LS3. PAL encoding transcript LOC107413687 showed upregulation when LS3 vs LS1, LS4 vs LS1, LS4 vs LS3, but no significant difference expression in LS3 vs LS2 and LS4 vs LS2. Benzaldehyde served as a key intermediate in the process of benzenoid compounds synthesis (Aragüez & Valpuesta Fernández, 2013). In *Prunus mume*, cytochrome P450s and short-chain dehydrogenases, reductases might regulate the production of benzaldehyde and benzyl alcohol (Wang, Song, et al., 2022).

Acetyl-CoA is the most abundant Co enzyme A in the fruit, functions as key intermediate for the synthesis of aroma compounds (Espino-Díaz et al., 2016) participating in multiple metabolic processes including the isoprenoid pathway, fatty acid synthesis, and the tricarboxylic acid cycle. These pathways produce intermediate metabolites such as acetic acid, aldehydes, ketones, and esters. The enzyme acetyl-CoA *C*-acetyltransferase (EC:2.3.1.9) catalyzed acetyl-CoA production, and the candidate transcript (LOC107415786) showed upregulation through the ripening. Pyruvate was another key intermediate for aroma compounds synthesis, metabolized via Pyruvate kinase (EC:2.7.1.40), whose encoding transcript LOC107430461 showed up regulation during jujube ripening.

### 3.6 Multivariate statistical analysis and integrative correlation network

**Fig. 2:**
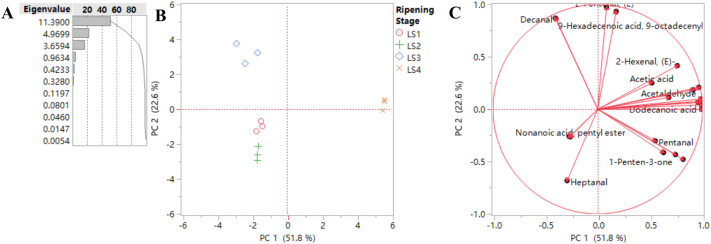
Principal Component Analysis based on semi-quantification of volatile compounds (A, Eigenvalue; B, score plot of first two principal components; C, loading plot of different variances).

To gain a systemic overview of the volatile dynamics and to identify key biomarkers during jujube fruit ripening, multivariate statistical analyses were employed. Principal Component Analysis (PCA), an unsupervised method, revealed a clear separation and ripening trajectory of the fruit samples from stages LS1 to LS4 along the principal component 1 (PC1) and principal component 2 (PC2), which accounted for 74.4 % of the total variance (Fig. 2B). The PCA analysis based on volatile compounds showed that the jujube Li fruits at LS1 and LS2 were similar, but they were distinguished from LS3 and LS4.

According to Pearson correlation analysis (Table 5), the strongly negative correlations between ADH activity and key aldehydes represent one of the most significant findings of this study. The correlation coefficients of −0.733 (hexanal), −0.661 ((*E*)-2-nonenal), −0.575 (benzaldehyde), and −0.545 ((*E*)-2-hexenal) with ADH activity provide robust statistical evidence for a metabolic bottleneck at the aldehyde-to-alcohol conversion step. This phenomenon appears to be cultivar-specific to jujube cv. Li, as other fruits like apple (Espino-Díaz et al., 2016) and pear (Chen et al., 2020) efficiently convert aldehydes to their corresponding alcohols. The downregulation of ADH genes (*LOC107421625*, *LOC107421626*, *LOC107426564*) observed in our transcriptome data aligns with the enzymatic bottleneck. The differential correlations between LOX activity and various aldehydes, the correlation with (E)-2-hexenal (*r* = 0.443) suggests this compound is a direct LOX pathway product; and the weaker correlation with hexanal (*r* = 0.148) may indicate additional regulatory steps or competing metabolic fates. The moderate positive correlation between AAT activity and hexanal (*r* = 0.602) is paradoxical, as AAT typically utilizes alcohols rather than aldehydes as substrates. This correlation may indicate feedback regulation where aldehyde accumulation signals the need for ester production. The absence of significant esters despite measurable AAT activity strongly supported the hypothesis that alcohol substrate availability, rather than AAT capacity, limited ester formation in jujube fruits.

**Table 5:** Pearson correlation analysis between LOX pathway enzymes activity and volatile compounds detected in jujube cv. Li.

	LOX	ADH	AAT
Acetaldehyde	0.056	−0.800**	−0.236
Acetic acid	−0.076	−0.510	−0.381
1-Penten-3-one	0.326	−0.413	0.739**
Pentanal	−0.085	−0.305	0.281
2-Pentenal, (E)-	0.501	0.405	−0.626*
Hexanal	0.148	−0.733**	0.602*
2-Hexenal, (E)-	0.443	−0.545	−0.234
Heptanal	−0.092	0.314	0.710**
2,4-Hexadienal, (E,E)-	0.428	−0.619*	0.204
Benzaldehyde	0.402	−0.572	0.206
Hexanoic acid	0.680*	0.404	−0.499
Benzyl Alcohol	0.472	−0.536	0.099
2-Octenal, (E)-	−0.095	−0.349	0.453
Decanal	0.359	0.783**	−0.645*
2-Nonenal, (E)-	0.382	−0.661*	0.238
n-Decanoic acid	0.445	−0.588*	0.181
5,9-Undecadien-2-one, 6,10-dimethyl-	0.396	−0.623*	0.179
Dodecanoic acid	0.397	−0.612*	0.185
Nonanoic acid, pentyl ester	−0.637*	−0.430	−0.363
Octanoic acid, octyl ester	−0.648*	−0.453	−0.330
n-Hexadecanoic acid	0.225	−0.700*	−0.090
9-Hexadecenoic acid, 9-octadecenyl ester, (Z,Z)-	0.339	0.784**	−0.651*

Note: *p < 0.05; **p < 0.01;

**Fig. 3:**
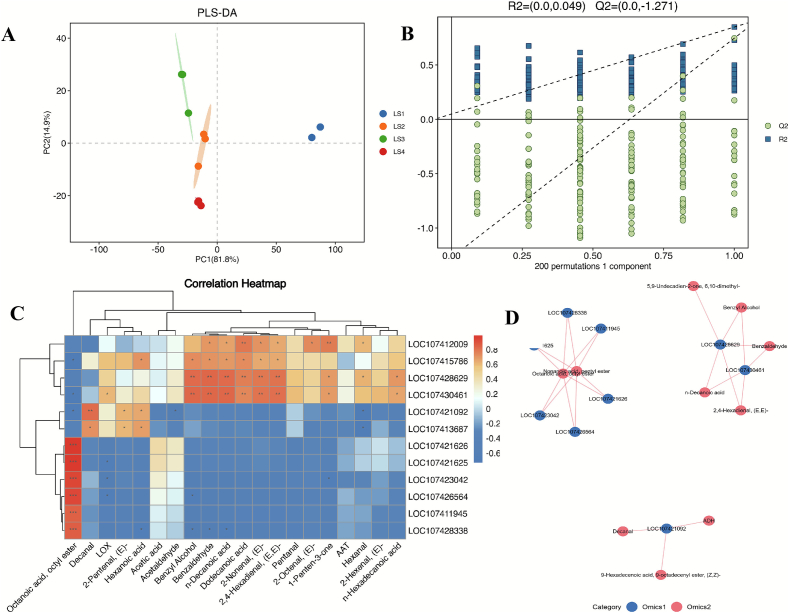
Multivariate statistical analysis of candidate transcripts and co-expression correlation network analysis. (A) PLS-DA and (B) OPLS-DA score plots based on the 12 candidate transcripts expression involved in aroma biosynthesis across the four ripening stages. (C) Correlation heatmap between the candidate transcripts and the volatile profiles, LOX pathway enzymes activity (*r* > 0.8, *p* < 0.05). (D) Co-expression correlation network of the candidate transcripts and key volatile compounds (r > 0.8, *p* < 0.05).

PLS-DA model (Fig. 3. A), constructed from the candidate transcripts expression dataset, achieved a clear separation of the four ripening stages, with the first two components explaining an exceptionally high cumulative variance of 96.7 %. Permutation testing (*n* = 200) confirmed the statistical significance of the model (R^2^ = 0.049, Q^2^ = −1.271), with the original model parameters significantly exceeding those obtained from permuted data (*p* < 0.05). This validates the robustness of the observed separation pattern.

The co-expression network, which integrated transcriptomic data of candidate genes with metabolomic profiles, transformed our understanding from a linear pathway to a dynamic interaction network. This analysis provided direct mechanistic support for several key hypotheses. First, the strong positive correlations between the Aldehyde dehydrogenase-encoding transcript LOC107428629 and a cluster of aldehyde compounds (e.g., hexanal, 2-nonenal, 2,4-Hexadienal) visually affirm the central role of this enzyme in channeling substrates into the aldehyde synthesis branch. Pyruvate kinase encoding transcript LOC107430461, which involved in fatty acids synthesis was strongly correlation with the aldehydes similar as transcript LOC107428629. Second, and critically, the network reveals the ADH-mediated metabolic bottleneck not as a mere absence of activity, but as a systemic disconnect at the molecular level. The negative correlations observed among ADH transcripts (LOC107421625, LOC107421626 and LOC107426564) and the volatile compounds; moreover, the corresponding alcohol compounds missing which provided compelling evidence that the transcriptional down regulation of these transcripts is a primary cause for the failure to convert accumulating aldehydes to their corresponding alcohols, the finding that aligns perfectly with our low ADH enzyme activity assays. There was a strongly positive correlation between transcript LOC107415786 (encoding Acetyl-CoA Acyltransferase) and n-decanoic acid, dodecanoic acid, (*E*)-2-nonenal, that may relate to fatty acids synthesis. Linoleate 9 s-lipoxigenase encoding with transcript Loc107412009 had a positive correlation with (*E*)-2-nonenal, this confirmed with the semi-quantification content of (*E*)-2-nonenal during ripening. LOX enzyme encoding transcript LOC107428338 was strongly positive correlation with octanoic acid octyl ester, which similarly reported that the genes of LOX enzyme contributed to the production of esters (Zhang et al., 2011).

Furthermore, the network uncovered evidence of cross-talk between distinct metabolic pathways. The tight correlation module comprising Benzaldehyde, Benzyl Alcohol, and n-Decanoic acid suggests a previously unappreciated coordination between the phenylpropanoid and fatty acid metabolic branches during ripening. This could be mediated by shared regulatory transcription factors or interconnected cofactor pools (e.g., NADPH), indicating that aroma biosynthesis is an integrated process rather than a collection of independent pathways.

## 4 Conclusion

This study provides comprehensive insights into the volatile biosynthesis pathways in *Ziziphus jujuba* cv. Li during fruit maturation through integrated GC–MS analysis, OAV evaluation, enzyme activity assays, and transcriptomic profiling. Three characteristic aroma-active compounds including hexanal, (E)-2-hexenal, and (E)-2-nonenal were identified as maturation stage-specific biomarkers. A key finding was the absence of LOX pathway-derived alcohols and esters across all developmental stages, despite increasing LOX activity. This, coupled with low ADH activity and the downregulation of ADH transcripts, indicates a metabolic bottleneck at the aldehyde-to-alcohol conversion step, which appears to be a distinctive feature of this jujube cultivar. Transcriptomic analysis revealed 12 candidate transcripts encoding critical enzymes, including acetyl-CoA acyltransferase, NAD-dependent aldehyde dehydrogenase, and linoleate 9S-lipoxygenase, whose expression patterns were temporally coordinated with volatile accumulation. Multivariate statistical analysis further strengthened these findings by demonstrating significant correlations between the expression of these candidate transcripts and the dynamic changes in the volatile profile.

The findings are based on a single jujube cultivar (cv. Li) and semi-quantitative volatile profiling. Notably, all data were derived from three biological replicates without technical replication, a design that prioritizes capturing biological variation across developmental stages. The key conclusion regarding the LOX pathway bottleneck is supported not by individual measurements but by consistent statistical trends across independent replicates and integrated data types (volatile compounds, enzyme activities, and gene expression). While technical replication could refine analytical precision, its absence does not undermine the identified biological patterns. Nevertheless, the proposed regulatory mechanisms require functional validation (e.g., through heterologous expression or gene silencing), and direct application to processed jujube products remains a focus for future research.

Notwithstanding these limitations, the elucidation of the ADH-mediated metabolic constraint provides novel insights into the regulation of the LOX pathway in fruits and addresses a significant knowledge gap in jujube volatile metabolism. The transcriptional regulators and candidate transcripts identified here offer a genetic foundation and potential targets for future metabolic engineering strategies. Subsequent research should focus on validating the function of these key transcripts and exploring their roles across different jujube cultivars, which would be a critical step toward ultimately customizing aroma profiles for quality improvement in jujube fruits.

## CRediT authorship contribution statement

**Lina Wang:** Writing – review & editing, Writing – original draft, Project administration, Funding acquisition. **Qiaoling Liu:** Writing – review & editing, Writing – original draft. **Chuan Chen:** Writing – original draft, Methodology, Data curation. **Rongfa Guan:** Writing – review & editing. **Peilong Sun:** Investigation.

## Declaration of competing interest

The authors declare that they have no known competing financial interests or personal relationships that could appeared to influence the work reported in this paper.

## Data Availability

Data will be made available on request.

## Appendix A Supplementary data

Supplementary material Image 1
