# Supplementary material for: Transcriptomic and biochemical insights into LOX pathway aroma biosynthesis during ripening of Ziziphus jujuba Mill. cv. Li
Source: Food Chem (Oxf). 2025 Dec 30;12:100347. doi: 10.1016/j.fochms.2025.100347 (PMC12810557; doi:10.1016/j.fochms.2025.100347)
Supplement: Supplementary file 1 — Supplementary material [file mmc1.docx]

***S.1 Differential gene expression analysis***

S.1.1 Differentially expressed genes distribution

According to **Fig. S1**, there were 4003, 6165, 9638, 393, 7307 and 6724 differentially expressed genes identified for the jujube Li fruits in the four ripening stages: LS2 (half-red stage) vs. LS1(white stage), LS3 (whole-red stage) vs. LS1, LS4(post-ripening stage) vs. LS1, LS3 vs. LS2, LS4 vs. LS2, LS4 vs. LS3. Among them, the highest differential genes number occurred between ripening stage LS4 and LS1, including both up-regulated and down-regulated genes. The smallest differential genes number occurred between LS3 and LS2 (**Fig. S1**).

S.1.2 GO functional annotation and KEGG enrichment analysis of differentially expressed genes

To elucidate the functional implications of differentially expressed genes (DEGs), Gene Ontology (GO) enrichment analysis was performed across three ontological categories: biological process (BP), cellular component (CC), and molecular function (MF). The results revealed significant enrichment of DEGs in cellular component-related terms during pairwise comparisons of distinct maturity stages. As illustrated in the corresponding figure, the majority of DEGs were clustered in level 2 classifications of biological processes and cellular components. According to **Fig. S2**, key enriched cellular component terms included cytosol (GO:0005829), mitochondria (GO:0005739), chloroplast stroma (GO:0009570), chloroplast envelope (GO:0009941), and thylakoids (GO:0009579). Within biological processes, DEGs showed pronounced enrichment in photosynthesis (GO:0015979) and heat response (GO:0009408), while molecular function annotations were dominated by unfolded protein binding (GO:0051082) and catalytic activity (GO:0003824). In previous study, Huang et al. found that at three maturity stages of pepper, the functional subclasses including 22 biological processes, 13 cellular components and 10 molecular functions were enriched (Huang et al., 2023).

We investigated the top 20 pathways with the highest gene enrichment of KEGG pathway. DEGs with varying stages of maturation showed significant enrichment. Based on **Fig. S3**, it was shown that DEGs were predominantly enriched in pyruvate metabolism, glycolysis/gluconeogenesis, glutathione metabolism, fatty acid biosynthesis, fatty acid degradation in LS2 vs LS1, the fruits were developed at this period. When LS3 compared with LS1, the DEGs were enriched in pyruvate metabolism, glycolysis/gluconeogenesis, α-linolenic acid metabolism, amino sugar and nucleotide sugar metabolism, purine metabolism; and it was shown that DEGs were mainly enriched in protein processing in endoplasmic, ribosome, and some amino acids metabolisms for LS4 vs LS1. Moreover, in **Fig. S3 C, E, F**, pairwise comparisons of later maturity stages (LS3 vs LS2, LS4 vs LS2, LS4 vs LS3) showed consistent enrichment in amino acid metabolism pathways. Given that amino acids and fatty acids serve as critical precursors for aroma compounds (Huang et al., 2023; Yuan et al., 2020). these metabolic shifts likely underlie the dynamic changes in volatile profiles observed during fruit ripening.

References

Huang, C., Sun, P., Yu, S., Fu, G., Deng, Q., Wang, Z., & Cheng, S. (2023). Analysis of Volatile Aroma Components and Regulatory Genes in Different Kinds and Development Stages of Pepper Fruits Based on Non-Targeted Metabolome Combined with Transcriptome. *International Journal of Molecular Sciences*, *24*(9), 7901. https://doi.org/10.3390/ijms24097901

Yuan, F., Yan, J., Yan, X., Liu, H., & Pan, S. (2020). Comparative transcriptome analysis of genes involved in volatile compound synthesis in blueberries (Vaccinium virgatum) during postharvest storage. *Postharvest Biology and Technology*, *170*, 111327. https://doi.org/10.1016/j.postharvbio.2020.111327


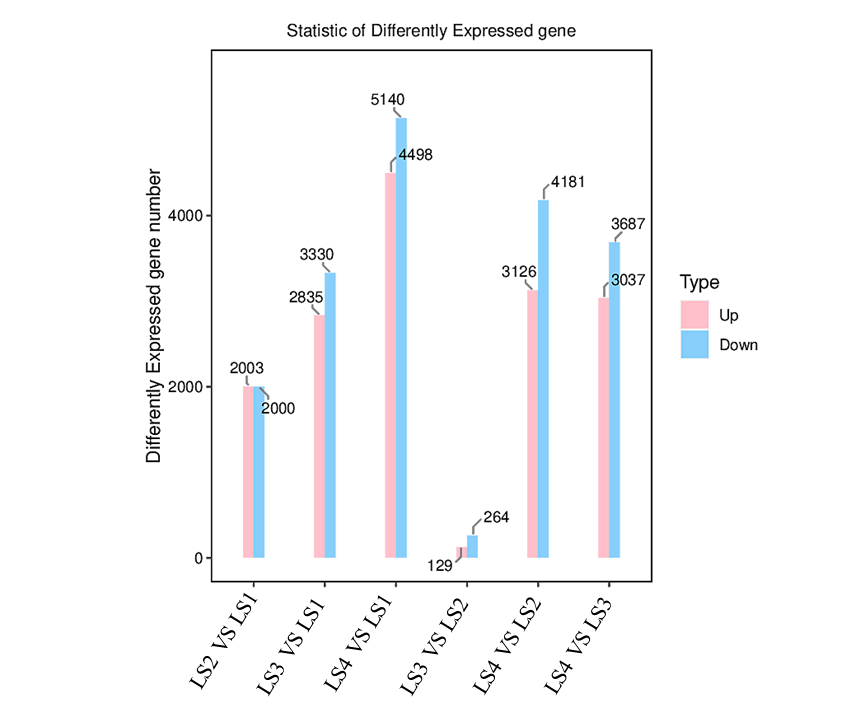


Fig. S1. Comparison of different expressed genes between each two ripening stages


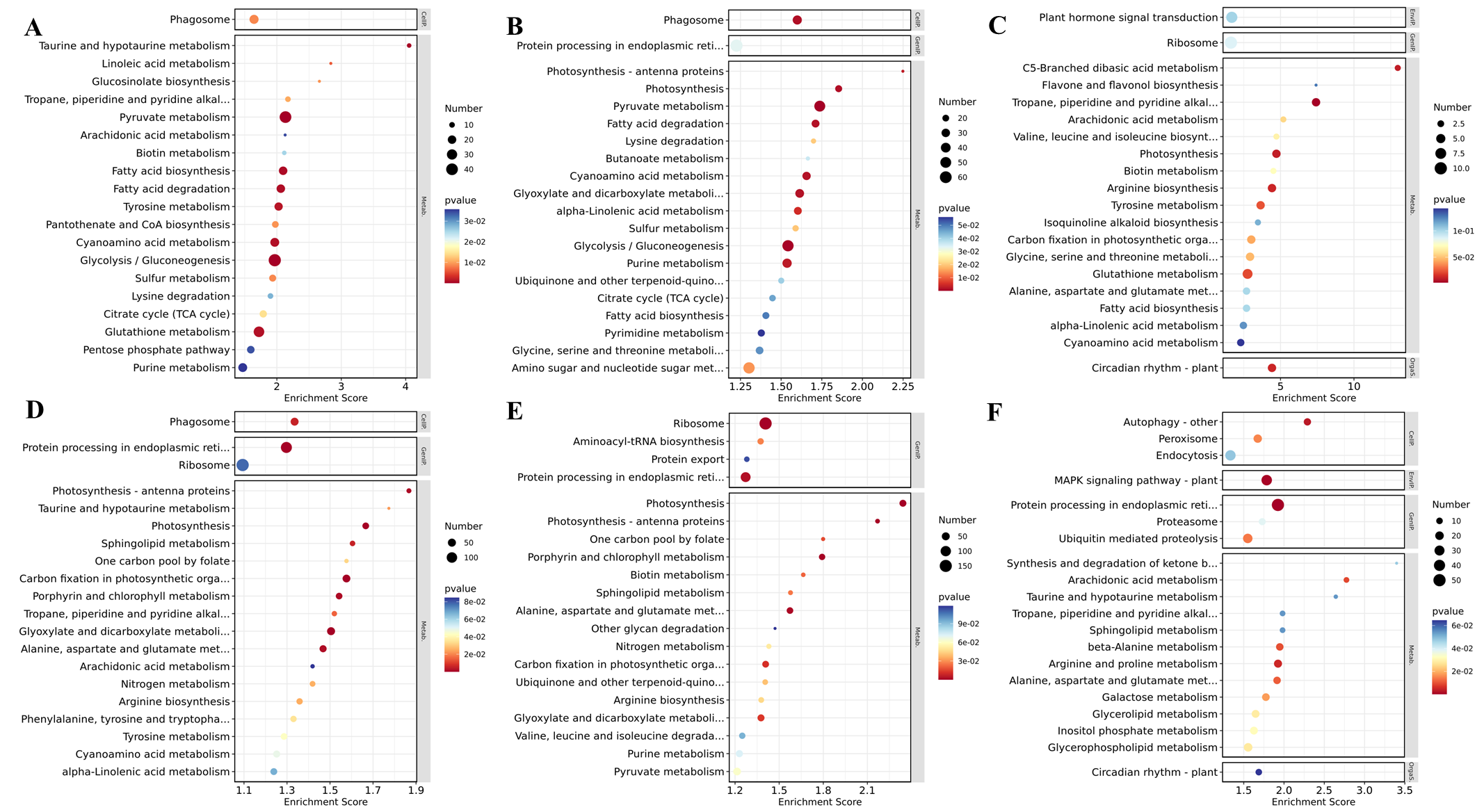


Fig. S2. GO classification compared among different ripening stages (A: LS2 vs LS1; B: LS3 vs LS1; C: LS3 vs LS2; D: LS4 vs LS1; E: LS4 vs LS2; F: LS4 vs LS3)


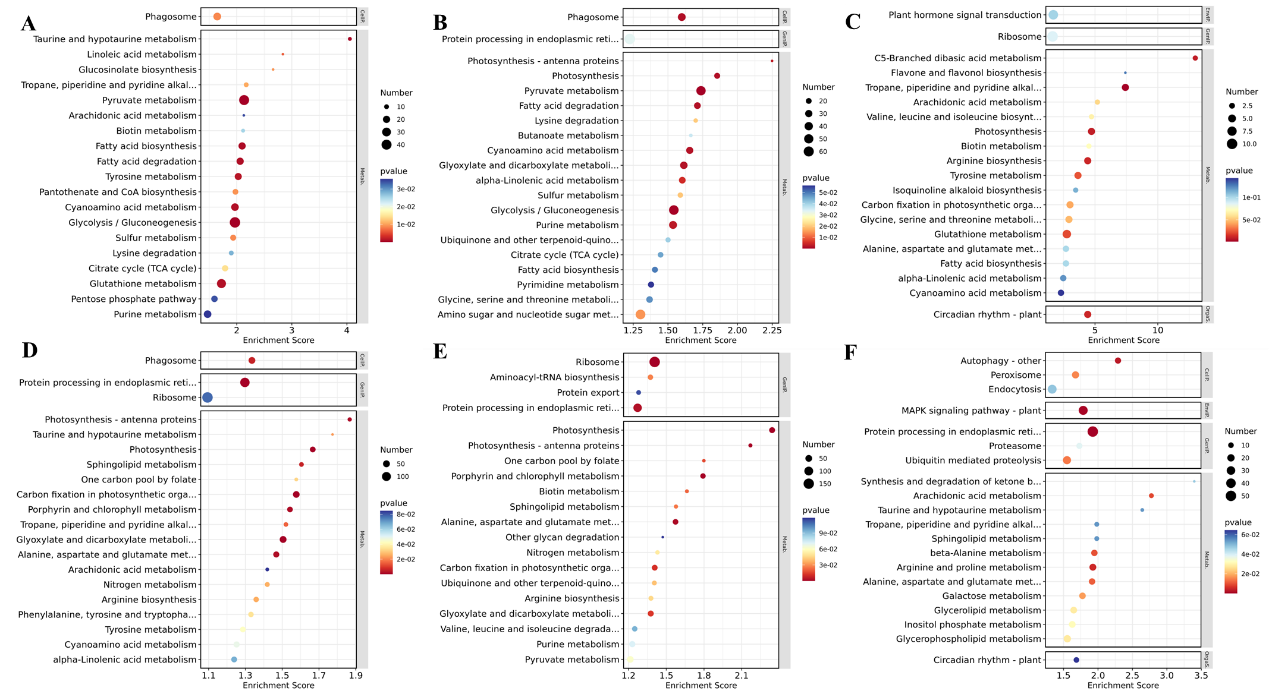


Fig. S3. KEGG enrichment top 20 from different ripening stages (A: LS2 vs LS1; B: LS3 vs LS1; C: LS3 vs LS2; D: LS4 vs LS1; E: LS4 vs LS2; F: LS4 vs LS3)
